# Brute Force Composition Scanning with a CALPHAD Database to Find Low Temperature Body Centered Cubic High Entropy Alloys

**DOI:** 10.3390/e20120911

**Published:** 2018-11-29

**Authors:** T. P. C. Klaver, D. Simonovic, M. H. F. Sluiter

**Affiliations:** Department of Materials Science and Engineering, Delft University of Technology, 2628 CD Delft, The Netherlands

**Keywords:** high entropy alloy, bcc, phase stability, CALPHAD, composition scanning

## Abstract

We used the Thermo-Calc High Entropy Alloy CALPHAD database to determine the stable phases of AlCrMnNbTiV, AlCrMoNbTiV, AlCrFeTiV and AlCrMnMoTi alloys from 800 to 2800 K. The concentrations of elements were varied from 1–49 atom%. A five- or six-dimensional grid is constructed, with stable phases calculated at each grid point. Thermo-Calc was used as a massive parallel tool and three million compositions were calculated, resulting in tens of thousands of compositions for which the alloys formed a single disordered body centered cubic (bcc) phase at 800 K. By filtering out alloy compositions for which a disordered single phase persists down to 800 K, composition ‘islands’ of high entropy alloys are determined in composition space. The sizes and shapes of such islands provide information about which element combinations have good high entropy alloy forming qualities as well as about the role of individual elements within an alloy. In most cases disordered single phases are formed most readily at low temperature when several elements are almost entirely excluded, resulting in essentially ternary alloys. We determined which compositions lie near the centers of the high entropy alloy islands and therefore remain high entropy islands under small composition changes. These island center compositions are predicted to be high entropy alloys with the greatest certainty and make good candidates for experimental verification. The search for high entropy islands can be conducted subject to constraints, e.g., requiring a minimum amount of Al and/or Cr to promote oxidation resistance. Imposing such constraints rapidly diminishes the number of high entropy alloy compositions, in some cases to zero. We find that AlCrMnNbTiV and AlCrMoNbTiV are relatively good high entropy alloy formers, AlCrFeTiV is a poor high entropy alloy former, while AlCrMnMoTi is a poor high entropy alloy former at 800 K but quickly becomes a better high entropy alloy former with increasing temperature.

## 1. Introduction

High entropy alloys (HEAs) are at present a very active field of research within metallurgy. The vast number of possible compositions promises a very broad range of properties. While the vast majority of (near) equi-atomic combinations of alloying elements lead to alloys with poor properties, the small fraction of combinations with good properties still provides very promising prospects, spurring very active research in this area.

Originally, HEAs were defined as alloys with five or more principal elements in (near) equi-atomic amounts, which form a single disordered phase on a simple crystal lattice. Configurational entropy was thought to be the main stabilizing factor, though it was soon shown that other factors can be more important, see e.g., [1]. More recently, the focus of attention has widened. More alloys that are not (near) equi-atomic have been investigated [2]. Carbon and/or nitrogen have been deliberately introduced to steer ferritic/austenic stability and to form finely dispersed carbides and/or nitrides to improve mechanical properties, see e.g., [3]. Compositions are chosen to deliberately create multi-phase materials that have better mechanical properties [4,5]. Stacking fault energies and relative phase stabilities in multi-phase materials are engineered to induce TRIP and/or TWIP deformation mechanisms [6,7,8,9,10,11,12]. Despite the extensive research effort on HEAs, the number of true HEAs found is still rather limited [13]. The vast majority of compositions lead to the formation of alloys with very brittle phases, like Laves and sigma phases [14]. Even many of the compositions that lead to alloys with good properties for applications are not truly HEAs at lower temperature. These alloys (sometimes referred to as compositionally complex alloys) may be HEAs just below the solidification temperature, but at lower temperature their equilibrium state includes additional phases [15,16]. They often have good low temperature properties thanks to the sluggish formation of additional phases, which allows the disordered single phase to persist as a meta-stable state at lower temperature.

In this work we focus on finding HEAs that retain their single disordered phase down to relatively low temperature, consisting in part of elements that promote oxidation resistance (Al up to high temperature, Cr up to intermediate temperature in environments free of water vapour). The number of non-equi-atomic composition variations with five or more elements is so large that experimental testing, even with modern high-throughput screening using samples with composition gradients, is no longer feasible. Computationally however, using CALPHAD databases to determine the stable phases as a function of temperature on a fine grid in the composition space is possible. For the six element alloys AlCrMnNbTiV and AlCrMoNbTiV, and five element alloys AlCrFeTiV and AlCrMnMoTi, and their constituent alloys, we determined in a five (four) dimensional composition space where the ‘islands’ of low temperature HEA stability are located, i.e., for which compositions a single disordered phase remains stable down to low temperature. Apart from determining islands of low temperature HEA stability we also determine where the ‘centers’ of the islands are, i.e., which compositions remain HEAs under small compositional changes. These compositions are also likely to have some margin against the inevitable error inherent in the CALPHAD method, see e.g., the mismatches in the comparison between CALPHAD predicitons and experimental results drawn up by Saal et al. [15]. The island centre compositions are predicted to be low temperature HEAs with the greatest certainty and are good candidates for experimental verification. Apart from selecting compositions corresponding to the centers of islands of HEA stability, constraints can be imposed. For example, minimum amounts of Al and/or Cr can be required to promote oxidation resistance. Also, alloys can be selected for a narrow solidification temperature range to limit segregation during solidification.

The outline of this paper is as follows: in Section 2 we provide details on our computational approach. In Section 3 we first explain our choice of the five and six element alloys we investigated and present results of a simple composition optimization for these alloys. We then present results of convergence testing of the concentration step size used in brute force scanning of the composition space for these alloys. After that, we look at the overall HEA forming qualities of the alloys and the roles that individual elements play in them through binary element projections. Finally, we present results about the islands of HEA stability for our alloys, without and with constraints for minimum concentrations of certain elements. Conclusions are reported in Section 4.

## 2. Computational Details

The Thermo-Calc (TC) implementation of the CALPHAD method was used to calculate stable phases. The TC high entropy alloy v2.1 database (TCHEA2.1 [17,18]) was used within TC v2017b or 2018a, run under linux. The TCHEA2.1 database contains data for the elements Al, C, Co, Cr, Cu, Fe, Hf, Mn, Mo, N, Nb, Ni, Re, Ru, Si, Ta, Ti, V, W and Zr. For these elements, full information on all binary systems and 135 ternary systems is included, as well as partial information from another 308 ternary systems. Equilibrium data for some of the elements (including Fe) is available only for ~500 °C and above. To avoid the hazards of extrapolation, our calculations apply to the temperature range 800–2800 K. Below 800 K diffusion is exceedingly sluggish in transition metal HEAs, so that equilibrium calculations are in any case more applicable to the higher temperature ranges. We found that calculations over a continuous temperature range with TCHEA2.1 enter into infinite loops every few dozen compositions, making automated high-throughput calculations ineffective. Also, results are at times calculated over incomplete temperature ranges. Calculations did not go into infinite loops when calculated with a different TC database (SSOL) or when data was calculated at discrete temperatures rather than continuously over a temperature range. Hence, we calculated data with TCHEA2.1 every 50 K in the 800–2800 K range (41 temperatures).

We employed a high throughput approach that is in some ways similar to the high-throughput method used by Senkov et al. [19,20]. In their extensive study, the Pandat implementation of the CALPHAD method was used to calculate the equilibrium phases for over 100,000 equi-atomic alloys. Here we determine equilibrium phases for a large number of non-equi-atomic compositions for four alloys. We used the Console.sh command line interface within TC to run typically ~100 calculations in parallel on single cpu cores of a computing cluster. The calculation of the stable phases and their fractions at 41 temperatures takes less than a minute on one cpu core, allowing throughput of a few thousand compositions per core per day. For this work we calculated 3 million compositions in total. While e.g., using a genetic algorithm to find HEA compositions [21], possibly in combination with a constraint satisfaction algorithm [22] or performing a targeted search that optimizes an objective function (e.g., narrow solidification temperature range or single disordered phase stability down to low temperature) under constraints [23] are approaches that are all far less computationally demanding, using TC as a high throughput tool is not much limited by the required cpu time or the disk space required to store input and output files. Analysis can be time consuming if it is done post hoc in serial over hundreds of thousands of output files. Analysis should ideally be included right after each TC calculation so that it is carried out in parallel, either using external tools or the TC_Python module.

## 3. Results and Discussion

### 3.1. Selection of Alloys, Extending the HEA Temperature Range

HEAs containing Al, Cr and Ti are rather likely to have a bcc crystal structure. Pure Cr has a bcc crystal structure and while Ti has an hcp structure at room temperature, it assumes a bcc structure above 1155 K. While pure Al has an fcc crystal lattice, it is known to promote the bcc structure in transition metal based HEAs [24]. The work by Senkov et al. [19,20] reported both five element and six element bcc HEAs (Tables 14 and 15 in [19]). TCHEA2.1 did not confirm all bcc HEAs predicted in [22], but several six element HEAs containing Al and Cr, including AlCrMnNbTiV and AlCrMoNbTiV, were confirmed to be HEAs. AlCrMnNbTiV is predicted to be a single disordered bcc phase from ~1550–1750 K, AlCrMoNbTiV from ~1200–2100 K. According to TCHEA2.1 the five element HEA AlCrTaTiV starts to form a sigma phase just before solidification is complete. AlCrFeTiV is predicted to be a disordered single bcc phase from ~1050–1800 K, AlCrMnMoTi from ~1150–1800 K. We focused our work on AlCrMnNbTiV, AlCrMoNbTiV, AlCrFeTiV and AlCrMnMoTi.

**Figure 1:**
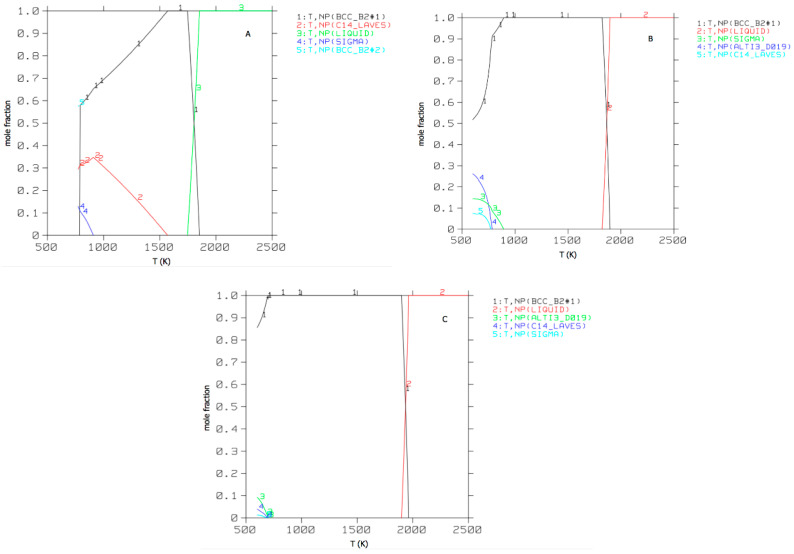
Phase fractions as a function of temperature for (**A**) equi-atomic AlCrMnNbTiV (**B**) Al_18_Cr_10_Mn_13_Nb_12_Ti_21_V_26_ (**C**) Al_15_Cr_12_Mn_17_Nb_3_Ti_10_V_43_.

For practical applications of these four series of alloys, it is preferable that the temperature at which other (brittle) phases appear is decreased and the amount of other phases formed is reduced. A simple way to achieve this is to determine what the composition of the disordered bcc phase and alternate phases is at a lower temperature, where multiple phases have formed. If the alternate phases were removed, the remaining bcc phase then forms a HEA at the lower temperature. This was tried for multiple iterations for AlCrMnNbTiV, see Figure 1.

Obviously, altering the concentrations of the individual elements within a HEA can be very effective in maintaining the HEA to a lower temperature and reducing the amount of alternate phases once they start to form. However, this way of strengthening the HEA character of an alloy produces a HEA that at low temperature is on the boundary of the HEA single phase region and the two or more phase region containing undesirable secondary phases.

The smallest change in composition in some directions already leads to the formation of secondary phases. In order to find an alloy that is a HEA ‘with margin to spare’, we want to find the compositions that remain HEAs under all small composition changes.

### 3.2. Convergence Testing for Scanning Part of the Composition Space

Scanning all possible five and six element alloy compositions at fine 1% increments requires going through more compositions than is feasible. In order to limit the number of compositions required, we limit the portion of the composition space that we cover and for that limited part of the composition space, we conduct convergence tests of the concentration increment, to see how fine a mesh is required. We limit the part of the composition space by requiring that no element in a HEA should be a majority constituent, i.e., the concentration of any element should be <50 atom%. Within the selected part of the composition space, atom percentages are varied from 1 to 49% for all but one element and the concentration of the last element is set to reach 100% in total. If the concentration of the last element has to be negative or larger than 50%, the composition is rejected. For five/six element alloys, each element has the role of ‘filler-up’ once and that of ‘independent variable’ four/five times. It should be noted that while the independent and filler-up elements have the same concentration increment, the possible concentrations of the filler-up element are shifted compared to those of the other elements. For example, the composition closest to a binary alloy has 49% of one element, 1% for four elements, leaving 47% for the filler-up element. Thus with a 4% concentration increment, the independently varied elements have concentrations of 49, 45, … 5, 1% while the filler-up element has concentrations of 47, 43, …, 7, 3%. Thus the possible element concentrations of the independently varied and filler-up elements are on sub-grids that have the same spacing but are shifted from each other. Hence a 4% concentration increment will result in some element concentrations being only 2% apart. Following the scheme outlined above, the numbers of compositions for five and six element alloys are as shown in Table 1.

**Figure 2:**
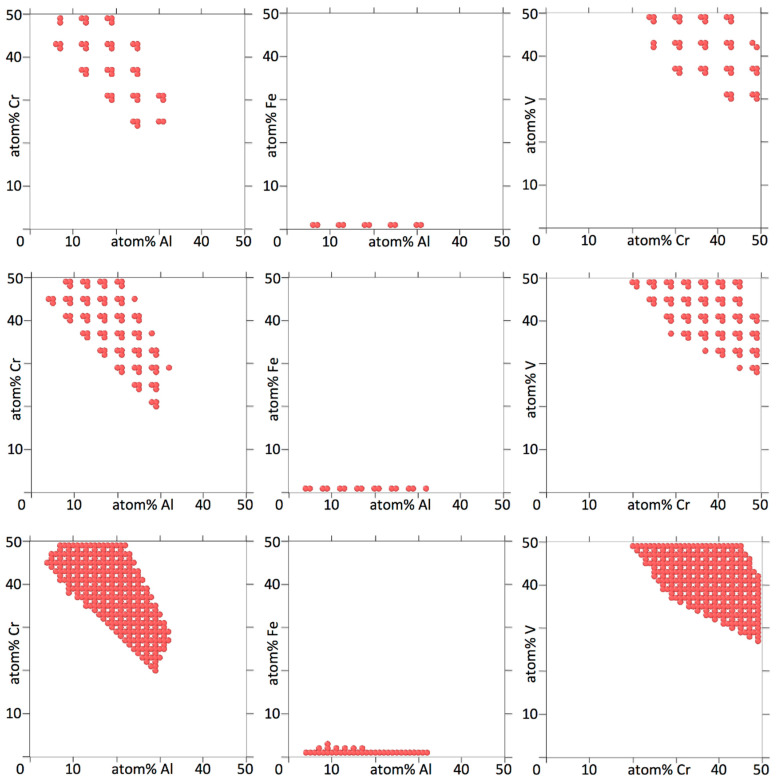
Two-dimensional projections for Al_a_Cr_b_Fe_c_Ti_d_V_1-a-b-c-d_, showing at which concentrations for two elements the alloy forms a bcc HEA at 800 K. Three concentration dimensions are flattened out to arrive at the two-dimensional projection. The concentrations of the three elements not shown can be any one or multiple combinations, i.e., a circle indicates that for the corresponding concentration of the two elements shown, there is at least one and in most cases there are many combinations of concentrations of the other three elements for which the alloy forms a HEA at 800 K. The concentration increments in the top, middle and bottom figures are 6, 4 and 2%, respectively.

**Figure 3:**
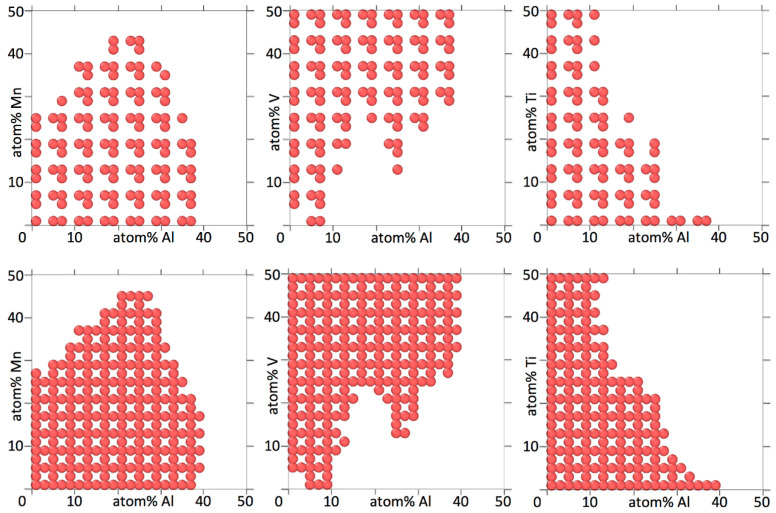
Two-dimensional projections for Al_a_Cr_b_Mn_c_Nb_d_Ti_e_V_1-a-b-c-d-e_, showing at which concentrations for two elements the alloy forms a bcc HEA at 800 K. Four concentration dimensions are flattened out to arrive at the two-dimensional projection. The concentrations of the four elements not shown are as explained in the caption of Figure 2. The concentration increments in the top and bottom figures are 6 and 4%, respectively.

The results we are most interested in are the shapes of low temperature islands of HEA stability. The convergence tests should therefore determine how much these vary with the concentration spacing. We show a number of two-dimensional projections for Al_a_Cr_b_Fe_c_Ti_d_V_1-a-b-c-d_ in Figure 2 and for Al_a_Cr_b_Mn_c_Nb_d_Ti_e_V_1-a-b-c-d-e_ in Figure 3.

In the small sampling of projections in Figure 2 and Figure 3 there are only single islands of HEA stability, there are no small separate islands. Also, the islands are solid without holes in them. Generally the size of the islands is many times larger than the concentration spacing. The concentration spacing therefore only influences the outer edges of the islands. At a coarser spacing, some detail of the shapes of outer edges of the islands is lost, but the overall shapes of the islands are preserved. This means that for the cases shown, a relatively modest number of compositions on a coarse grid in composition space already provide most information about islands of low temperature HEA stability.

### 3.3. The Different Roles of Alloying Elements

**Figure 4:**
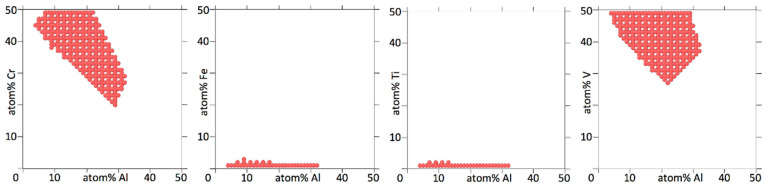

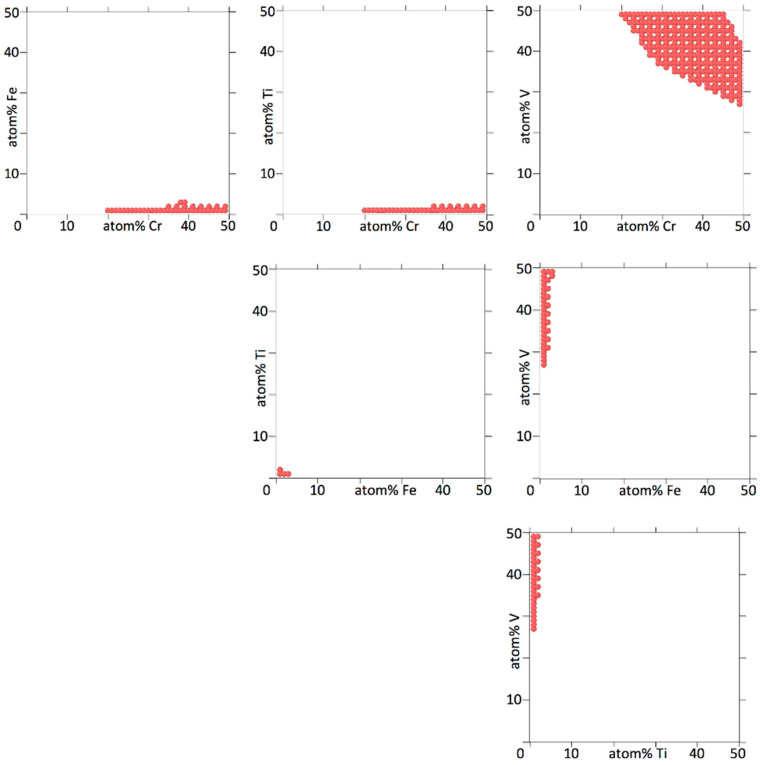
Two-dimensional projections for Al_a_Cr_b_Fe_c_Ti_d_V_1-a-b-c-d_, showing at which concentrations for two elements the alloy forms a bcc HEA at 800 K. Three concentration dimensions are flattened out to arrive at the two-dimensional projection. The concentrations of the three elements not shown are as explained in the caption of Figure 2. The concentration increments are 2%.

**Figure 5:**
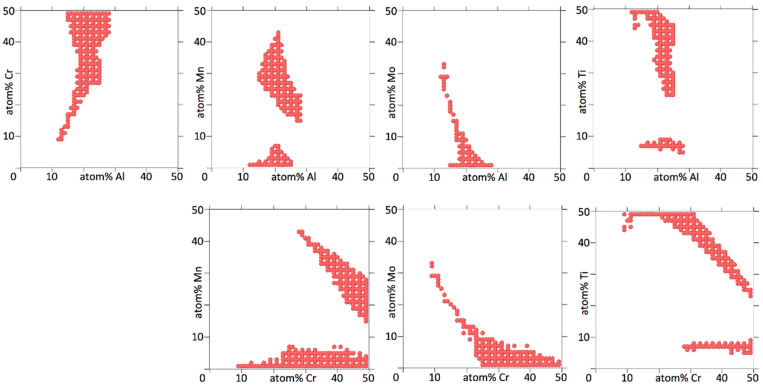

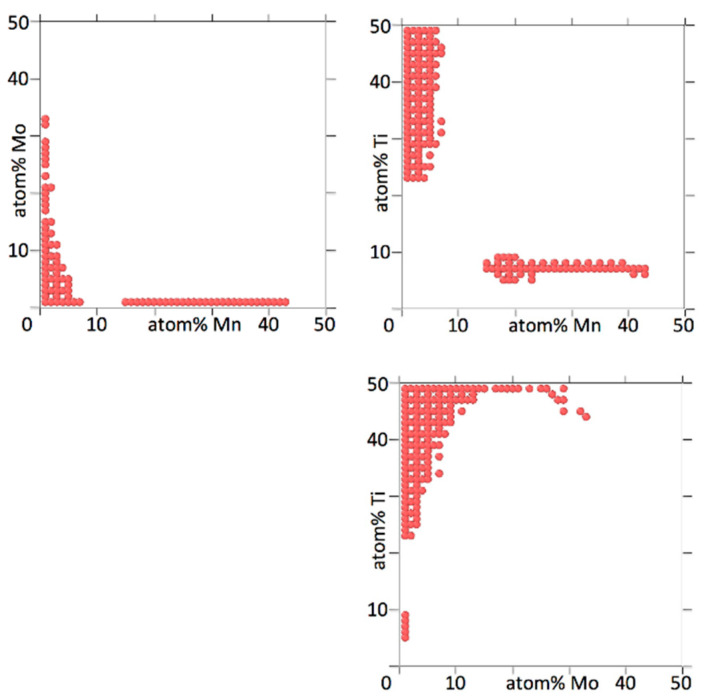
Two-dimensional projections for Al_a_Cr_b_Mn_c_Mo_d_Ti_1-a-b-c-d_, showing at which concentrations for two elements the alloy forms a bcc HEA at 800 K. Three concentration dimensions are flattened out to arrive at the two-dimensional projection. The concentrations of the three elements not shown are as explained in the caption of Figure 2. The concentration increments are 2%.

**Figure 6:**
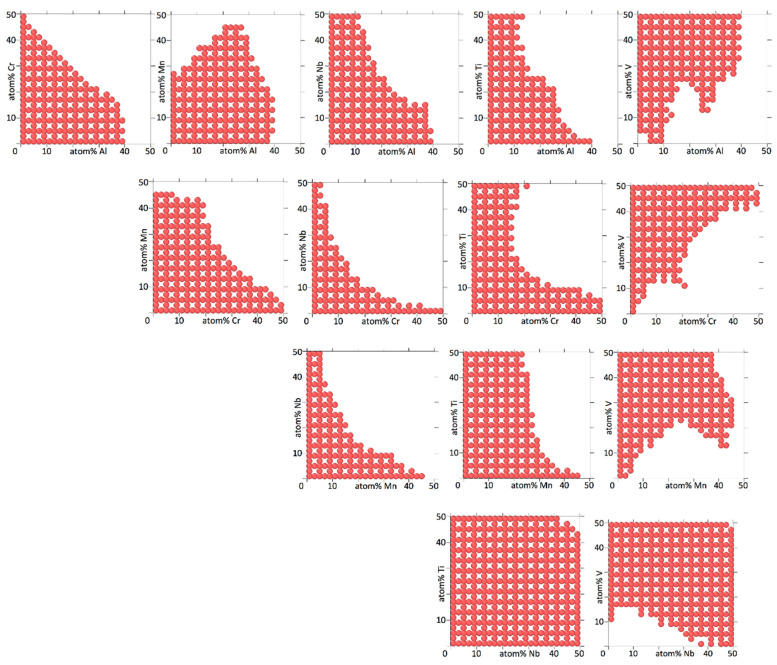

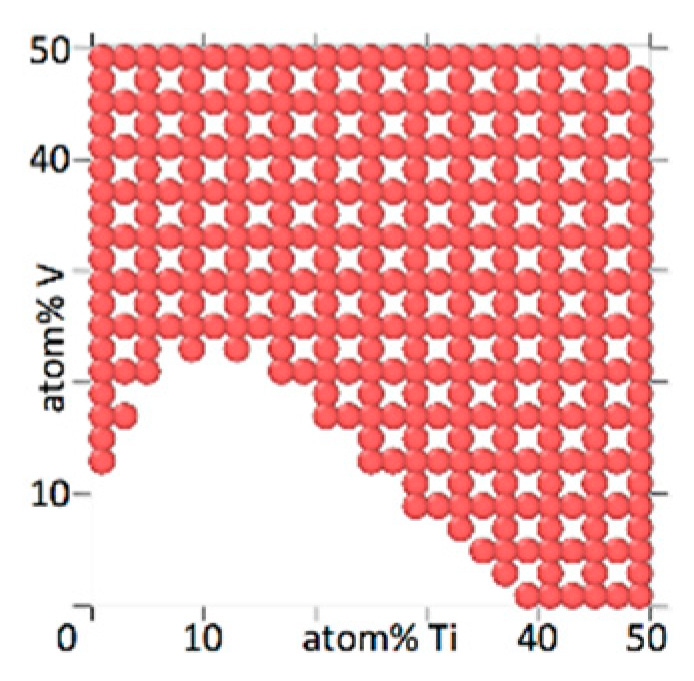
Two-dimensional projections for Al_a_Cr_b_Mn_c_Nb_d_Ti_e_V_1-a-b-c-d-e_, showing at which concentrations for two elements the alloy forms a bcc HEA at 800 K. Four concentration dimensions are flattened out to arrive at the two-dimensional projection. The concentrations of the four elements not shown are as explained in the caption of Figure 2. The concentration increments are 4%.

**Figure 7:**
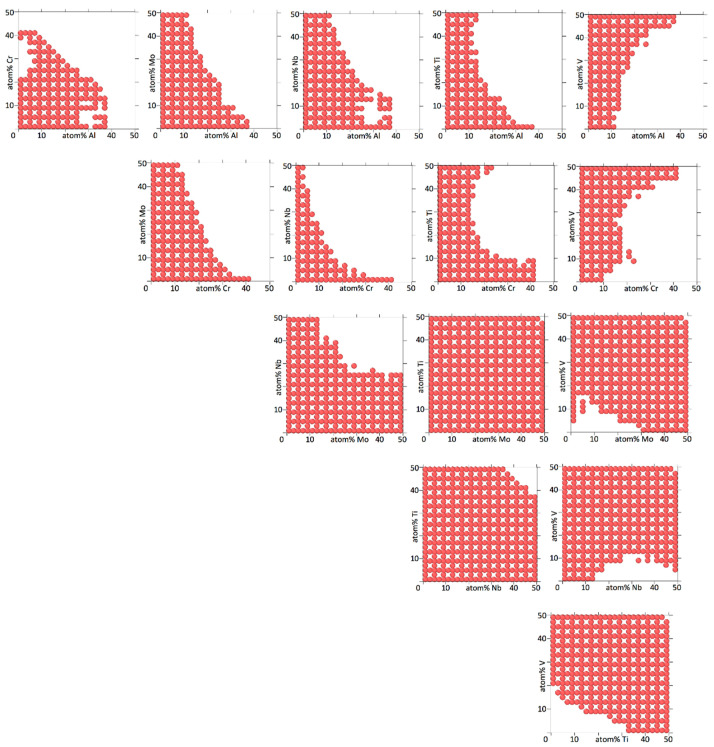
Two-dimensional projections for Al_a_Cr_b_Mo_c_Nb_d_Ti_e_V_1-a-b-c-d-e_, showing at which concentrations for two elements the alloy forms a bcc HEA at 800 K. Four concentration dimensions are flattened out to arrive at the two-dimensional projection. The concentrations of the four elements not shown are as explained in the caption of Figure 2. The concentration increments are 4%.

Figure 4, Figure 5, Figure 6 and Figure 7 show binary projections as in Figure 2 and Figure 3 for all possible binary combinations in our alloys.

In interpreting Figure 4, Figure 5, Figure 6 and Figure 7, it is worth pointing out that a lot of information is left out of the two-dimensional projections. What appears to be a single island may in fact consists of several separate islands in the dimension perpendicular to the projection (which contains all the information of the other elements than the two being shown), that overlap into a single island when shown as a two-dimensional projection.

Figure 4, Figure 5, Figure 6 and Figure 7 show that the various elements in the four alloys play distinct roles. On the one hand, Fe and Ti in AlCrFeTiV hardly participate in forming a HEA. Single disordered bcc phases in AlCrFeTiV can form, but they are essentially ternary alloys, without Fe or Ti. On the other hand, Mo and Ti in AlCrMoNbTiV can form HEAs with the other elements at any combination of concentrations. In between these two extremes, a variety of other behaviors can be observed. HEA islands that cover part of the two-element projections may extend mutually over the full 0–50% range for both elements or over the full range for one element but part of the range for the other, or over part of the range for both elements. A minimum concentration of the two elements can be required, indicated by a lack of circles around the origin, such as for TiV in AlCrMnNbTiV, see Figure 6. The HEA island may be formed under an inversely proportional line, such as for CrNb in AlCrMnNbTiV, see Figure 6. The maximum percentage of one element as a function of the other may not follow a monotonous line, there may be minima and maxima such as for AlMn and AlV in AlCrMnNbTiV, see Figure 6. V in AlCrMnNbTiV in particular gives many minima and maxima in the two-dimensional projections in Figure 6. There may even be an archipelago of separate islands of stability, as is the case with the thin, stretched-out islands for AlCrMnMoTi, see Figure 5. Islands are seen to feature a great variety of shapes, including bays, peninsular outcroppings and satellite islands, see AlCr, AlNb and CrV in AlCrMoNbTiV, Figure 7. Contrary to the results of convergence testing in Section 3.2, some of these features would be lost if the calculations were carried out on a coarser grid.

Overall, the two six element alloys appear to be more promising candidates for forming low temperature HEAs than the two five element alloys. For AlCrMnNbTiV and AlCrMoNbTiV 17,830 (3.8%) and 17,289 (3.7%) out of 473,382 compositions sampled were single phase HEAs at 800 K. For AlCrFeTiV and AlCrMnMoTi only 356 (0.041%) and 785 (0.091%) out of 862,750 compositions sampled were single phase HEAs at 800 K. In Figure 6 and Figure 7 on average 64% and 67% of the grid points of the two-dimensional projections for AlCrMnNbTiV and AlCrMoNbTiV have circles on them, while in Figure 4 and Figure 5 these percentages are only 6.8% and 9.4% for AlCrFeTiV and AlCrMnMoTi. The supplementary material contains the list of compositions calculated and for each composition, whether that composition is a HEA at 800 K or not and what the phases and phase fractions are at 800 K for the four alloy systems.

### 3.4. Temperature Dependence of HEA Stability

**Figure 8:**
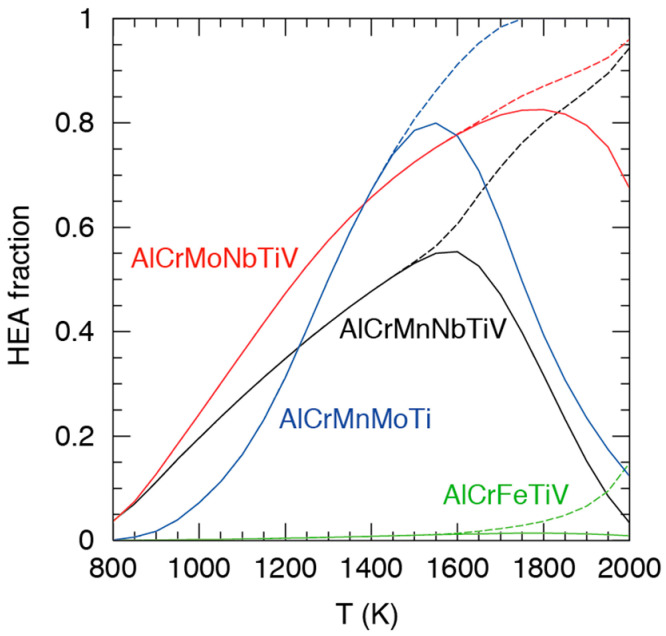
Fractions of alloy compositions for which a HEA is formed, as a function of temperature. Solid curves represent the number of HEAs as a fraction of all compositions, including those that are (partly) molten. Dashed curves represent the number of HEAs as a fraction of compositions for which the alloys are still completely solid.

Figure 8 shows the fraction of alloy compositions for which a HEA is formed as a function of temperature. 

**Figure 9:**
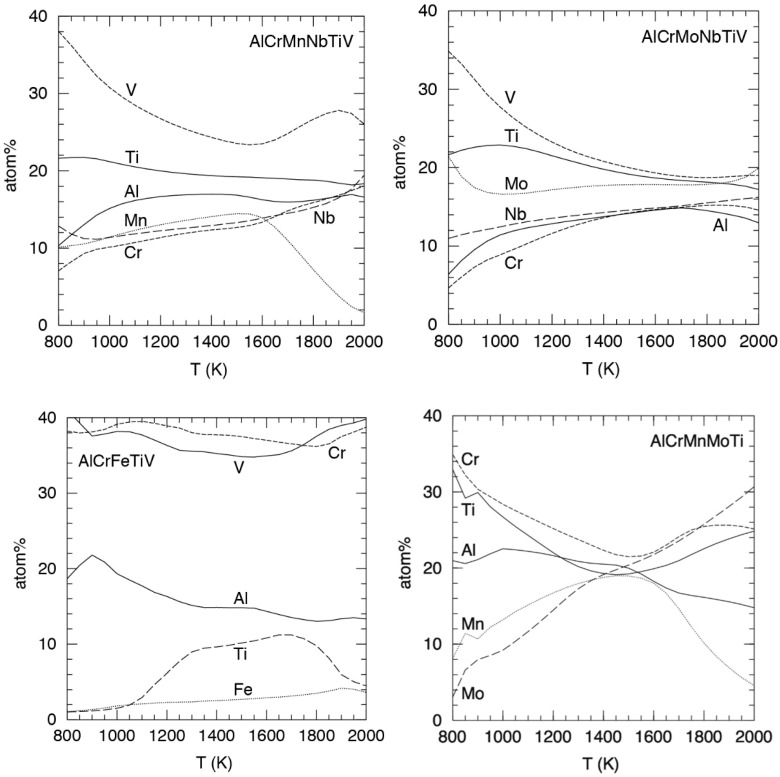
Average atom percentages of elements as a function of temperature, averaged over those compositions that form HEAs.

Figure 8 shows that both six element alloys are strong HEA formers, with 4% of compositions being HEAs at 800 K and the HEA fraction of fully solid alloys reaching over 90% at 2000 K. The much lower melting temperature of Mn (1519 K) compared to Mo (2896 K) increases the fraction of (partly) molten alloys at 2000 K but it does not greatly increase the onset of melting, since melting is likely to occur first for compositions rich in low-melting metals like Al. Also, alloys with little Mn or Mo are almost the same. In contrast to the six element alloys, AlCrFeTiV obviously has poor HEA forming qualities. AlCrMnMoTi is in between the six element alloys and AlCrFeTiV, with a very low fraction of HEAs at 800 K, but the fraction rapidly increases with temperature, surpassing that of the six element alloys and reaching 100% at 1750 K. Figure 9 shows the average concentrations of individual elements in HEAs as a function of temperature.

It is perhaps surprising to observe that in AlCrMnNbTiV and AlCrMnMoTi, Mn is the element that is the most reduced in concentration at higher temperatures, while pure Mn has a far higher melting temperature (1519 K) than pure Al (933 K). At present we are not able to explain this. For AlCrFeTiV the average composition shown in Figure 9 does not actually lie inside a HEA island for most of the temperature range. For the other three alloys the average concentrations shown in Figure 9 do lie inside HEA islands for all but a few of the lowest temperatures.

It should be noted that our calculations assume thermodynamic equilibrium and therefore homogenous phases. During solidification usually concentration gradients in the solid state develop so that our results may deviate from experimentally prepared materials.

### 3.5. HEA Island Centers

The center of a HEA island is here defined as the HEA composition that is furthest removed from any composition that is not a HEA. The distance between the island center and the closest non-HEA composition defines a body around the island center that contains a subset of the compositions that form the island. The size of the body indicates how much the concentrations of any element(s) can be varied from the island center while the alloy still remains a HEA. The island center—closest non-HEA distance can be calculated as the Euclidian distance (in which case the body is a high-dimensional spheroid) or Manhattan distance (in which case the body is a high-dimensional polyhedron, with a larger volume than the spheroid). Since we allow concentrations up to 50%, an island center may be close to 50% for one or two elements. Therefore it needs to be decided what to do with compositions on grid points on or outside the 50% boundary, for which there is no data. On the one hand, since the vast majority of compositions are not HEAs, it could be assumed that any composition on or outside the 50% boundary is not a HEA. This means that the sphere or polyhedron around the center must lie entirely within the 50% boundaries. On the other hand, if there is a part of an island of HEA stability bordering the 50% boundary, it is reasonable to assume that the island would not end abruptly at the 50% boundary but extend some distance beyond it as well. Therefore it could be argued that the center of the island needs to lie within the 50% boundary, but that part of the sphere or polyhedron may lie outside it. These two scenarios represent extremes for the smallest and biggest possible spheres/polyhedra and specific cases will usually lie somewhere in between. We shall present results for both scenarios, where all compositions at or beyond 50% are assumed to be non-HEAs (‘boundary_on’) or where compositions at or beyond 50% are assumed to be HEAs (‘boundary_off’). For the former scenario, non-HEA composition data points are added (i.e., defined, not calculated with TC) for all compositions where one or two elements have a 50% concentration. As an example, Table 2 shows the island center(s) composition for AlCrMnNbTiV.

The alternative, equally valid island center compositions indicated by the asterisks in Table 2 are compositions like 3, 1, 1, 25, 21, 49% or 1, 1, 3, 21, 25, 49% Al, Cr, Mn, Nb, Ti, V.

Under boundary_on condition, the Euclidian distance between the island center and the nearest non-HEA compositions is √102 = 10.1%. This is only a few times the concentration increment, hence the figure of 10.1% is not very precise. However, it does mean that the alloy will remain a HEA under limited composition changes. For example, if any one element is changed 9% in one direction and four other elements are changed 2% in the opposite direction and one element is changed 1% in the opposite direction, the resulting alloy should still be a HEA. Table 3 shows the island center compositions for all four of our HEAs and the distances to the closest non-HEA compositions.

**Figure 10:**
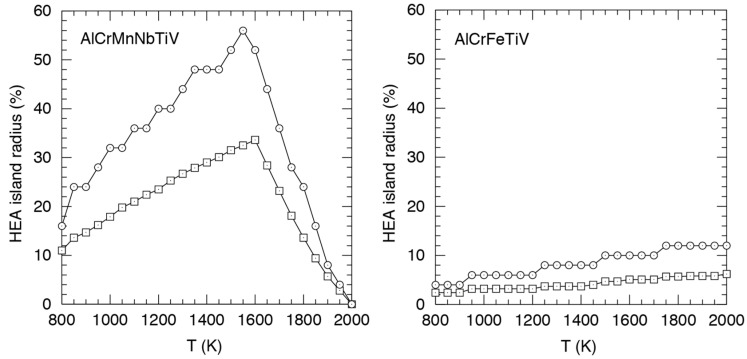
Euclidian (squares) and Manhattan (circles) radii of HEA islands around Al_1_Cr_1_Mn_1_Nb_25_Ti_31_V_41_ and Al_11_Cr_43_Fe_1_Ti_1_V_44_ under boundary_off condition. Radii are here defined as the distances between the island center and the nearest non-HEA composition. Only compositions *less* than a radius away from the island center are guaranteed to be HEAs.

As in Figure 4, Figure 5, Figure 6 and Figure 7, the compositions in Table 3 show that the six element alloys are much better HEA formers than the five element alloys at 800 K. The island radii for the five element alloys are so small that there are not really any HEA islands, just a few isolated HEA compositions, possibly with a very small number of their closest neighbor compositions. Finally in this section we show how HEA islands grow with temperature. Figure 10 shows the radii as a function of temperature for AlCrMnNbTiV and AlCrFeTiV.

Unsurprisingly, the island radius pattern for AlCrMnNbTiV in Figure 10 is rather similar to the pattern of the AlCrMnNbTiV HEA fraction shown for AlCrMnNbTiV in Figure 8.

### 3.6. HEA Compositions with Minimum Concentration Constraints

The compositions in Table 3 are all essentially ternary alloys, meaning they are not really conventional HEAs. For the six element alloys, the elements mostly absent from the island center compositions include Al and Cr. While oxidation resistance depends on more than just having significant amounts of Al and/or Cr present in alloys, their presence is an important enabling factor for oxidation resistance. We repeated our search for HEA islands of maximum size, but now under the condition that minimum amounts of Al and/or Cr are present in the alloys or that four or more elements must be present in a concentration equal or greater than 10%. Table 4 and Table 5 show HEA island center compositions and sizes determined under these constraints.

Table 4 and Table 5 show that the options for selecting HEAs with minimum Al and/or Cr content or four or more elements present in 10% or higher concentration are limited. Imposing such constraints decreases HEA island sizes, down to 0 when requiring that all elements have a 10% or higher concentration. AlCrMnNbTiV has the largest islands of HEA compositions that contain a high enough percentage of Al to promote oxidation resistance.

### 3.7. Melting Temperature Ranges

From a production point of view it is preferable to select alloys with narrow solidification temperature ranges in order to achieve solidification with minimal unmixing, or with unmixing on the smallest possible length scales. Since we only determine data at temperature intervals of 50 K, we can only roughly estimate solidification temperature ranges. Therefore, in Table 6 we list the number of 50 K spaced temperatures that fall within the solidification ranges.

Table 6 shows that alloy compositions that are a single phase HEAs at 800 K generally have rather narrow solidification ranges, in all cases six intervals of 50 K or less. On average the most promising HEA alloys from the AlCrMnNbTiV and AlCrMoNbTiV type have solidification ranges of just 1.27 and 3.28 50 K intervals. So it appears as if selecting compositions that are single phase HEAs at 800 K simultaneously also selects alloys that have desirable solidification behavior. However, the alloy with by far the narrowest solidification range, AlCrFeTiV, is also the poorest HEA former in our study. As in Section 3.4, the higher temperature results in this section have an extra deviation from experimental observations due to the artificial homogeneity and lack of any concentration gradients in our calculations.

## 4. Conclusions

We used the Thermo-Calc CALPHAD database to computationally investigate the HEA forming qualities between 800 and 2800 K of four alloys, AlCrMnNbTiV, AlCrMoNbTiV, AlCrFeTiV and AlCrMnMoTi. These alloys contain elements that provide oxidation resistance and were previously predicted to be HEAs at high temperature at equi-atomic compositions. Simple variations of the element concentrations away from being equi-atomic can already greatly extend the temperature range over which the alloys are HEAs. However, with a brute force compositions scanning approach, alloy compositions could be found that remain HEAs down to 800 K. By calculating the stable phases for these alloys on grids in five- or six-dimensional composition spaces, we were able to determine islands of low temperature HEA stability. Making binary alloy projections of these high-dimensional islands gives information about the overall HEA forming qualities of the alloys as well as about the roles of individual elements within the alloys. The HEA forming qualities of a combination of elements can also be gleaned from the percentage of compositions that form HEAs as a function of temperature. The compositions of the centers of the HEA islands remain HEAs under small composition changes and thus have some margin of error against inaccuracies in the TC HEA database. Applying our methodology to four alloys, we find that AlCrMnNbTiV and AlCrMoNbTiV are good HEA formers that have HEA islands of non-negligible size at 800 K and that these islands grow rapidly with increasing temperature. AlCrMnMoTi has very few HEA compositions at 800 K but rapidly develops them with increasing temperature. AlCrFeTiV is a poor HEA former at any temperature. For all alloys that have HEA islands, the island centre compositions correspond to what are essentially ternary alloys. Therefore these most interesting compositions are at best medium entropy alloys rather than high entropy alloys. The elements mostly absent from island centre compositions include Al and Cr for AlCrMnNbTiV and AlCrMoNbTiV. Alloys with these compositions thus lack elements that are important for oxidation resistance. Imposing constraints for minimal amounts of Al and/or Cr or four or more alloying elements with >10% concentration rapidly diminishes the number of available HEA compositions, though there are compositions that meet both the requirements of forming HEAs at 800 K and containing substantial amounts of Al and/or Cr. These requirements can be combined with the additional requirement of having a narrow solidification range. Alloy compositions around Al_25_Cr_7_Mn_25_Nb_1_Ti_1_V_41_ or Al_21_Cr_7_Mn_21_Nb_1_Ti_9_V_41_ offer the best compromise between these three different criteria, according to our CALPHAD predictions. Since CALPHAD predictions are sometimes at odds with experimental results [15], we propose these two compositions for experimental verification.

## Figures and Tables

**Table 1:** Numbers of compositions for five and six element alloys for different concentration spacings.

Concentration Spacing (%)	Compositions	
	Five elements	Six elements
6	13,530	74,412
4	60,905	473,382
2	862,750	2,114,580 *

* not calculated with TC in our study.

**Table 2:** HEA island center composition for AlCrMnNbTiV, determined with different distance and boundary criteria at 800 K. Also shown are the five non-HEA compositions closest to the island center. The elements concentration spacing is 4%. An asterix behind an island center composition indicates there are other island center compositions nearby that have an equally long distance to a nearest non-HEA composition.

	Distance and Boundary Criteria			
	Euclidean Distance		Manhattan distance	
	Boundary_on	Boundary_off	Boundary_on	Boundary_off
	% Al Cr Mn Nb Ti V			
island centre(s)	1 1 1 25 31 41	1 1 1 23 25 49	1 3 1 21 37 37 * 1 1 13 5 39 41 *	1 3 1 21 25 49 *
1st nearest non-HEA	0 0 0 22 28 50 10.1% from center	11 1 1 17 25 45 12.3% from center	1 11 1 21 29 37 7 1 5 5 41 41 16% from center	1 11 1 25 13 49 24% from center
2nd nearest non-HEA	0 0 0 21 29 50 10.2% from center	9 1 1 17 29 43 12.3% from center	1 11 1 21 33 33 7 1 9 1 41 41 16% from center	1 11 1 25 17 45 24% from center
3rd nearest non-HEA	0 0 0 23 27 50 10.2% from center	1 9 1 27 17 45 12.6% from center	1 11 1 21 37 29 7 1 13 1 41 37 16% from center	1 11 1 25 21 41 24% from center
4th nearest non-HEA	0 0 1 21 28 50 10.4% from center	1 9 1 27 21 41 12.6% from center	9 3 1 13 37 37 9 1 11 1 37 41 16% from center	1 1 11 25 21 41 24% from center
5th nearest non-HEA	0 0 1 22 27 50 10.4% from center	9 1 1 15 29 45 12.6% from center	9 3 1 17 33 37 9 1 11 5 33 41 16% from center	1 9 1 27 13 49 24% from center

**Table 3:** HEA island center(s) compositions, determined with different distance and boundary criteria at 800 K. Below each composition is the distance to the nearest non-HEA composition. The elements concentration spacing is 4% for the six element alloys and 2% for the five element alloys. An asterix behind an island center composition indicates there are other island center compositions nearby that have an equally long distance to a nearest non-HEA composition.

	Island Center(s) and Distance to Nearest Non-HEA Composition			
	Euclidean Distance		Manhattan Distance	
	Boundary_on	Boundary_off	Boundary_on	Boundary_off
Alloy	% Elements			
AlCrMnNbTiV	1 1 1 25 31 41 10.1%	1 1 1 23 25 49 12.3%	1 3 1 21 37 37 * 1 1 13 5 39 41 * 16%	1 3 1 21 25 49 * 24%
AlCrMoNbTiV	1 1 37 1 21 39 * 10.2%	1 1 37 1 13 47 * 11.3%	1 1 39 1 21 37 * 20%	1 1 35 1 13 49 * 24%
AlCrFeTiV	11 43 1 1 44 * 2.4%	11 43 1 1 44 * 2.4%	11 43 1 1 44 * 4%	11 43 1 1 44 * 4%
AlCrMnMoTi	22 33 1 1 43 * 3.7%	22 33 1 1 43 * 3.7%	22 33 1 1 43 * 6%	22 33 1 1 43 * 6%

**Table 4:** HEA island center(s) compositions, determined with different distance and boundary criteria at 800 K, under the constraint of having minimum amounts of Al and/or Cr present. Below each composition is the distance to the nearest non-HEA composition. The elements concentration spacing is 4% for the six element alloys and 2% for the five element alloys. An asterix behind an island center composition indicates there are other island center compositions nearby that have an equally long distance to a nearest non-HEA composition.

	Island Center(s) and Distance to Nearest Non-HEA Composition			
	Euclidean Distance		Manhattan Distance	
	Boundary_on	Boundary_off	Boundary_on	Boundary_off
Alloy, Constraint	% Al Cr Mn Nb Ti V			
AlCrMnNbTiV Al ≥ 15%	25 7 25 1 1 41 * 21 7 21 1 9 41 * 7.5%	25 1 23 1 1 49 9.8%	25 7 21 1 5 41 * 12%	21 5 21 1 5 47 * 16%
AlCrMnNbTiV Cr ≥ 15%	17 15 17 1 9 41 5.7%	11 17 13 1 9 49 * 6.3%	5 17 5 11 17 45 * 17 17 13 1 9 43 * 8%	11 17 13 1 9 49 * 12%
AlCrMnNbTiV Al + Cr ≥ 15%	25 7 25 1 1 41 * 21 7 21 1 9 41 * 7.5%	25 1 23 1 1 49 9.8%	25 7 21 1 5 41 * 7 9 5 17 21 41 * 12%	21 5 21 1 5 47 * 7 9 5 13 17 49 16%
AlCrMoNbTiV Al ≥ 15%	17 1 9 21 9 43 * 17 5 21 1 11 45 * 4.9%	15 1 5 21 9 49 * 6.3%	17 1 9 21 9 43 * 17 5 21 1 11 45 * 8%	15 1 5 21 9 49 * 12%
AlCrMoNbTiV Cr ≥ 15%	9 15 21 1 13 41 * 4.9%	15 17 1 5 13 49 13 17 11 1 9 49 * 4.9%	9 15 21 1 13 41 * 8%	15 17 1 5 13 49 13 17 11 1 9 49 * 8%
AlCrMoNbTiV Al + Cr ≥ 15%	9 7 29 1 13 41 * 6.3%	11 5 25 1 9 49 * 7.5%	9 7 29 1 13 41 * 12%	11 5 25 1 9 49 * 16%
AlCrFeTiV Al ≥ 15%	23 36 1 1 39 * 1.4%	23 36 1 1 39 * 1.4%	23 36 1 1 39 * 2%	23 36 1 1 39 * 2%
AlCrFeTiV Cr ≥ 15%	11 43 1 1 44 * 2.4%	11 43 1 1 44 * 2.4%	11 43 1 1 44 * 4%	11 43 1 1 44 * 4%
AlCrFeTiV Al + Cr ≥ 15%	11 43 1 1 44 * 2.4%	11 43 1 1 44 * 2.4%	11 43 1 1 44 * 4%	11 43 1 1 44 * 4%
AlCrMnMoTi Al ≥ 15%	22 33 1 1 43 * 3.7%	22 33 1 1 43 * 3.7%	22 33 1 1 43 * 6%	22 33 1 1 43 * 6%
AlCrMnMoTi Cr ≥ 15%	22 33 1 1 43 * 3.7%	22 33 1 1 43 * 3.7%	22 33 1 1 43 * 6%	22 33 1 1 43 * 6%
AlCrMnMoTi Al + Cr ≥ 15%	22 33 1 1 43 * 3.7%	22 33 1 1 43 * 3.7%	22 33 1 1 43 * 6%	22 33 1 1 43 * 6%

**Table 5:** HEA island center(s) compositions, determined with different distance and boundary criteria at 800 K, under the constraint of having four or more elements present in a ≥10% concentration. Below each composition is the distance to the nearest non-HEA composition. The elements concentration spacing is 4% for the six element alloys and 2% for the five element alloys. An asterix behind an island center composition indicates there are other island center compositions nearby that have an equally long distance to a nearest non-HEA composition.

	Distance and Boundary Criteria			
	Euclidean Distance		Manhattan Distance	
	Boundary_on	Boundary_off	Boundary_on	Boundary_off
Alloy, Constraint	% Al Cr Mn Nb Ti V			
AlCrMnNbTiV 4 elem. ≥ 10%	17 13 17 1 9 43 * 6.3%	11 1 9 17 13 49 17 11 17 1 9 45 * 5 11 1 13 21 49 * 5 1 11 13 25 45 * 7.5%	17 13 17 1 9 43 * 12%	17 11 17 1 9 45 * 16%
AlCrMnNbTiV 5 elem. ≥ 10%	17 13 17 1 11 41 6.3%	13 13 13 1 11 49 7.5%	17 13 17 1 11 41 12%	11 13 13 1 13 49 * 13 1 11 13 13 49 * 12%
AlCrMnNbTiV 6 elem. ≥ 10%	-	-	-	-
AlCrMoNbTiV 4 elem. ≥ 10%	11 5 29 1 13 41 * 6.3%	11 1 1 25 13 49 * 11 5 25 1 13 45 * 6.3%	11 1 29 5 13 41 * 12%	11 1 1 25 13 49 * 11 1 25 1 13 49 * 12%
AlCrMoNbTiV 5 elem. ≥ 10%	13 11 17 1 13 45 5.5%	13 11 17 1 13 45 5.7%	11 1 17 13 13 45 * 13 13 19 1 13 41 * 13 1 13 17 11 45 * 8%	11 1 13 13 13 49 * 13 13 17 1 13 43 * 13 11 29 1 17 29 8%
AlCrMoNbTiV 6 elem. ≥ 10%	-	-	-	-
AlCrFeTiV 4 elem. ≥ 10%	-	-	-	-
AlCrFeTiV 5 elem. ≥ 10%	-	-	-	-
AlCrMnMoTi 4 elem. ≥ 10%	16 17 1 17 49 * 1.4%	16 17 1 17 49 * 1.4%	16 17 1 17 49 * 2%	16 17 1 17 49 * 2%
AlCrMnMoTi 5 elem. ≥ 10%	-	-	-	-

**Table 6:** Number of 50 K spaced data points in the solidification temperature ranges of alloys.

Alloy	Average, All Compos.	Average Over HEA Compos. at 800 K	Average Over Non- HEA Compos. at 800 K	Max. of All Compos.	Max. of HEA Compos.
AlCrMnNbTiV	2.42	1.27	2.46	15	4
AlCrMoNbTiV	3.20	3.28	3.19	15	6
AlCrFeTiV	2.42	0.43	2.42	9	2
AlCrMnMoTi	4.21	1.68	4.21	15	4

## Supplementary Materials

The following are available online at http://www.mdpi.com/1099-4300/20/12/911/s1. The supplementary material contains the list of compositions calculated and for each composition whether that composition is a HEA at 800 K or not and what the phases and phase fractions are at 800 K for the four alloy systems.
